# Supplementary material for: A benchmark dataset for machine learning in ecotoxicology
Source: Sci Data. 2023 Oct 18;10:718. doi: 10.1038/s41597-023-02612-2 (PMC10584858; doi:10.1038/s41597-023-02612-2)
Supplement: Supplementary file 1 — Supplementary Information [file 41597_2023_2612_MOESM1_ESM.pdf]

A benchmark dataset for machine learning in ecotoxicology

Christoph Schür[1,†,*], Lilian Gasser[2,†], Fernando Perez-Cruz[2,4], Kristin Schirmer[1,3,5], and Marco Baity-Jesi[1]

[1]Eawag, Swiss Federal Institute of Aquatic Science and Technology, Dübendorf, Switzerland
[2]Swiss Data Science Center (SDSC), Zürich, Switzerland
[3]ETH Zürich: Department of Environmental Systems Science, Zürich, Switzerland
[4]ETH Zürich: Department of Computer Science, Zürich, Switzerland
[5]EPF Lausanne, School of Architecture, Civil and Environmental Engineering, Lausanne, Switzerland
[*]corresponding author: Christoph Schür (christoph.schuer@eawag.ch)
[†]these authors contributed equally to this work

SUPPLEMENTARY INFORMATION

1 Glossary

Table 1. A glossary of all columns in the dataset. The prefix of the column name identifies the feature category or source file, *e.g.,* column names starting with "test" originate from the ECOTOX tests file. The dataset contains modeling features as well as additional information. Only the features labelled "suitable for modeling" should be used for modeling. The others *must not* be used. Effective concentrations are labelled as targets. Only one target should be used and the others *must not* be used as modeling feature. *We advise modelers to investigate these features, *e.g.,* with a correlation analysis, before adding them to the model.

Column name	Description	Suitable for modeling
test_id	Unique identifier for an experiment (links with ECOTOX)	no
reference_number	Unique identifier for the original source (links with ECOTOX)	no
test_cas	CAS number of the chemical compound	no
test_location	Test location	no
test_exposure_type	Exposure type	yes
test_control_type	Control type	no
test_media_type	Media type	yes
test_application_freq_unit	Application frequency unit	no
test_organism_lifestage	Organism life stage	no
result_id	Unique identifier for each data point (links with ECOTOX)	no
result_effect	Effect group	no
result_endpoint	Endpoint	no
result_obs_duration_mean	Observation duration	yes
result_conc1_type	Exposure concentration type	yes
result_conc1_mean_op	Effective concentration operator	no
result_conc1_mean	Effective concentration value (in mg/L)	target
result_conc1_mean_mol	Effective concentration value (in mol/L)	target
media_ph_mean	Measured medium pH	yes
media_temperature_mean	Measured medium temperature (in °C)	yes
tax_all	Combination of all taxonomic levels	no
tax_name	Common species name	no
tax_class	Taxonomic class	no
tax_order	Taxonomic order	no
tax_family	Taxonomic family	no
tax_genus	Taxonomic genus	no
tax_species	Taxonomic species	no
tax_gs	Taxonomic genus and species	no
species_number	Species identifier (links with ECOTOX)	no
tax_group	Taxonomic group	no
tax_pdm_available	Boolean whether phylogenetic distance is available	no
tax_eco_climate	Ecology, climate zone	yes
tax_eco_ecozone	Ecology, ecozone	yes
tax_eco_food	Ecology, food class	yes
tax_eco_migrate5	Ecology, migratory behavior (5-level encoding)	yes
tax_eco_migrate2	Ecology, migratory behavior (2-level encoding)	yes
tax_lh_amd	Life history, life span (in d)	yes
tax_lh_lbcm	Life history, body length at birth (in cm)	yes
tax_lh_lpcm	Life history, body length at puberty (in cm)	yes
tax_lh_licm	Life history, ultimate body length (in cm)	yes
tax_lh_ri#/d	Life history, reproductive rate (in $\#/d$)	yes
tax_ps_ampv	Pseudo-data, energy conductance (in cm/d)	yes
tax_ps_ampkap	Pseudo-data, allocation fraction to soma	yes

Table 1 continued from previous page

Column name	Description	Suitable for modeling
tax_ps_amppm	Pseudo-data, volume-specific somatic maintenance cost (in $J/d \cdot cm^3$)	yes
result_conc1_mean_binary	Effective concentration category (more toxic/less toxic)	target
result_conc1_mean_log	Effective mass concentration value after a log10 transformation	target
result_conc1_mean_mol_log	Effective molar concentration value after a log10 transformation	target
chem_dtxsid	DSSTOX substance ID	no
chem_name	Name of chemical compound	no
test_cas_name	Concatenation of CAS number and name of chemical compound	no
chem_sf	Molecular formula	no
chem_mw	Molecular weight (in g/mol)	yes
chem_mp	Melting point (in °C)	yes
chem_ws	Water solubility (in mg/L)	yes
chem_ws_binary	Boolean whether water solubility is available	no
chem_dtxcid	DSSTOX compound ID	no
chem_inchi	InChI from DSSTox	no
chem_inchikey	InChIkey from DSSTox	no
chem_pcp_cid	PubChem compound ID	no
chem_pcp_inchi	InChI from PubChem	no
chem_pcp_inchikey	InChIkey from PubChem	no
chem_pcp_iupac_name	IUPAC name	no
chem_pcp_can_smiles	Canonical SMILES from PubChem	no
chem_pcp_fp	Collapsed PubChem fingerprint	yes
chem_pcp_heavy_atom_count	Number of heavy atoms, *i.e.,* not hydrogen	yes
chem_rdkit_clogp	Octanol-water partition coefficient	yes
chem_rdkit_can_smiles	Canonical SMILES from RDKit	no
chem_pcp_bonds_count	Number of bonds	yes
chem_pcp_doublebonds_count	Number of double bonds	yes
chem_pcp_triplebonds_count	Number of triple bonds	yes
chem_rings_count	Number of rings	yes
chem_OH_count	Number of OH groups	yes
chem_mol2vec_allowed	Boolean: compatible with mol2vec	no
chem_pka_median	Acid dissociation constant	yes
chem_MACCS_fp	Collapsed MACCS fingerprint	yes
chem_Morgan_fp	Collapsed Morgan fingerprint	yes
chem_ToxPrint_fp	Collapsed ToxPrint fingerprint	yes
chem_mol2vec[000-299]	300-dimensional mol2vec embedding	yes
chem_mordred_x	Mordred features	yes*
split_totallyrandom	Split data points totally at random (incl. cross-validation folds)	no
split_random	Split chemicals at random (incl. cross-validation folds)	no
split_occurrence	Split chemicals by occurrence (incl. cross-validation folds)	no
split_scaffold-murcko	Split chemicals by Murcko scaffold (incl. cross-validation folds)	no
split_scaffold-murcko-loo-0	Split chemicals by Murcko scaffold (only training and test set)	no
split_scaffold-murcko-loo-1	Split chemicals by Murcko scaffold (only training and test set)	no
split_scaffold-murcko-llo	Split chemicals by Murcko scaffold (only training and test set)	no
split_scaffold-generic	Split chemicals by generic scaffold (incl. cross-validation folds)	no
split_scaffold-generic-loo-0	Split chemicals by generic scaffold (only training and test set)	no
split_scaffold-generic-loo-1	Split chemicals by generic scaffold (only training and test set)	no
split_scaffold-generic-llo	Split chemicals by generic scaffold (only training and test set)	no

2 ECOTOX data

Definitions for ECOTOX effect groups as given in the ECOTOX term appendix:

- **Mortality (MOR):** Measurements and endpoints where the cause of death is by direct action of the chemical.
- **Physiology (PHY)/Intoxication (ITX):** Measurements and endpoints regarding basic activity in cells and tissues of plants or animals; includes four effect groups - injury, immunity, intoxication and general physiological response.
- **Growth (GRO):** Category encompasses measures of weight and length, and includes effects on development, growth and morphology.
- **Population (POP):** Measurements and endpoints relating to a group of organisms or plants of the same species occupying the same area at a given time.

2.1 Toxicity categories

The toxicity intervals given in Table 2 and shown for the chemicals in our dataset in Figure 3 are in accordance with EPA.

Table 2. EC50 intervals for the binary and multi-class toxicity classification used in Figure 3.

EC50 interval (mg/L)	Binary classification	Multi-class classification
$(-\infty, 10^{-1}]$	more toxic	very highly toxic
$(10^{-1}, 10^{0}]$	more toxic	highly toxic
$(10^{0}, 10^{1}]$	less toxic	moderately toxic
$(10^{1}, 10^{2}]$	less toxic	slightly toxic
$(10^{2}, +\infty)$	less toxic	non-toxic

2.2 Reference chronology

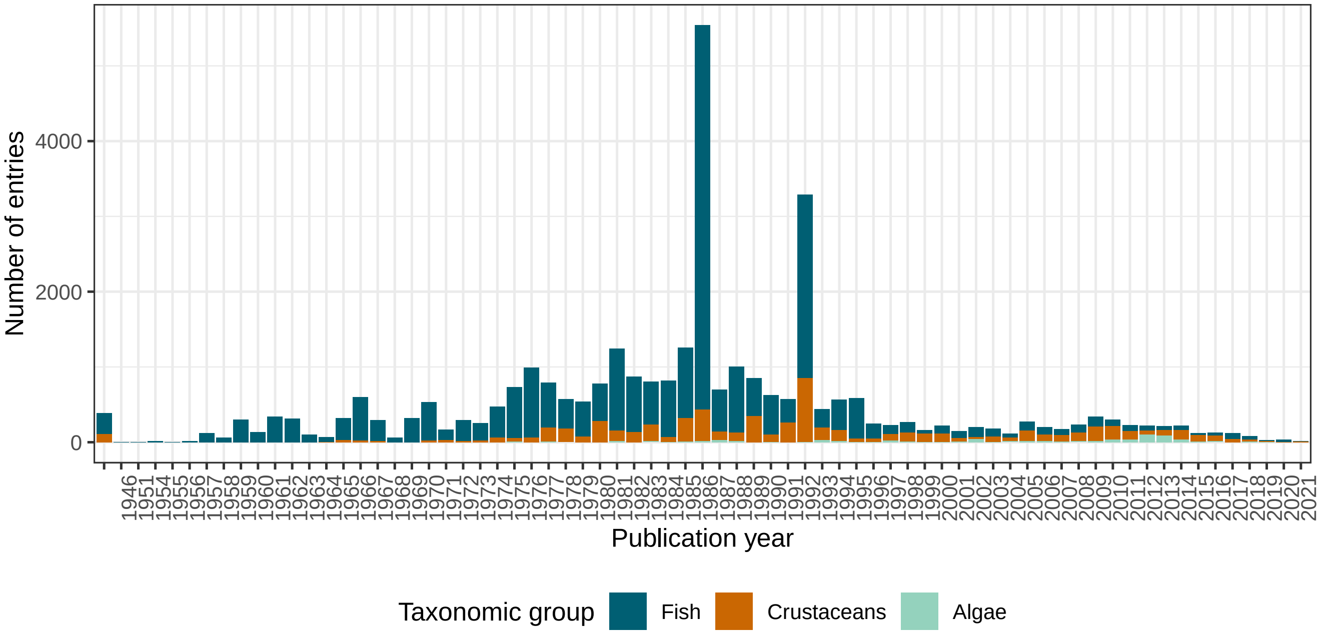

Figure 1. Chronology of the references related to the different taxonomic groups added to ECOTOX over time. The publication year refers to the year the original study was published. It is noteworthy that some references are not singular scientific studies, but entail whole databases, such as the US EPA "Pesticide Ecotoxicity Database (Formerly: Environmental Effects Database (EEDB))", added in 1992.

2.3 Experimental properties

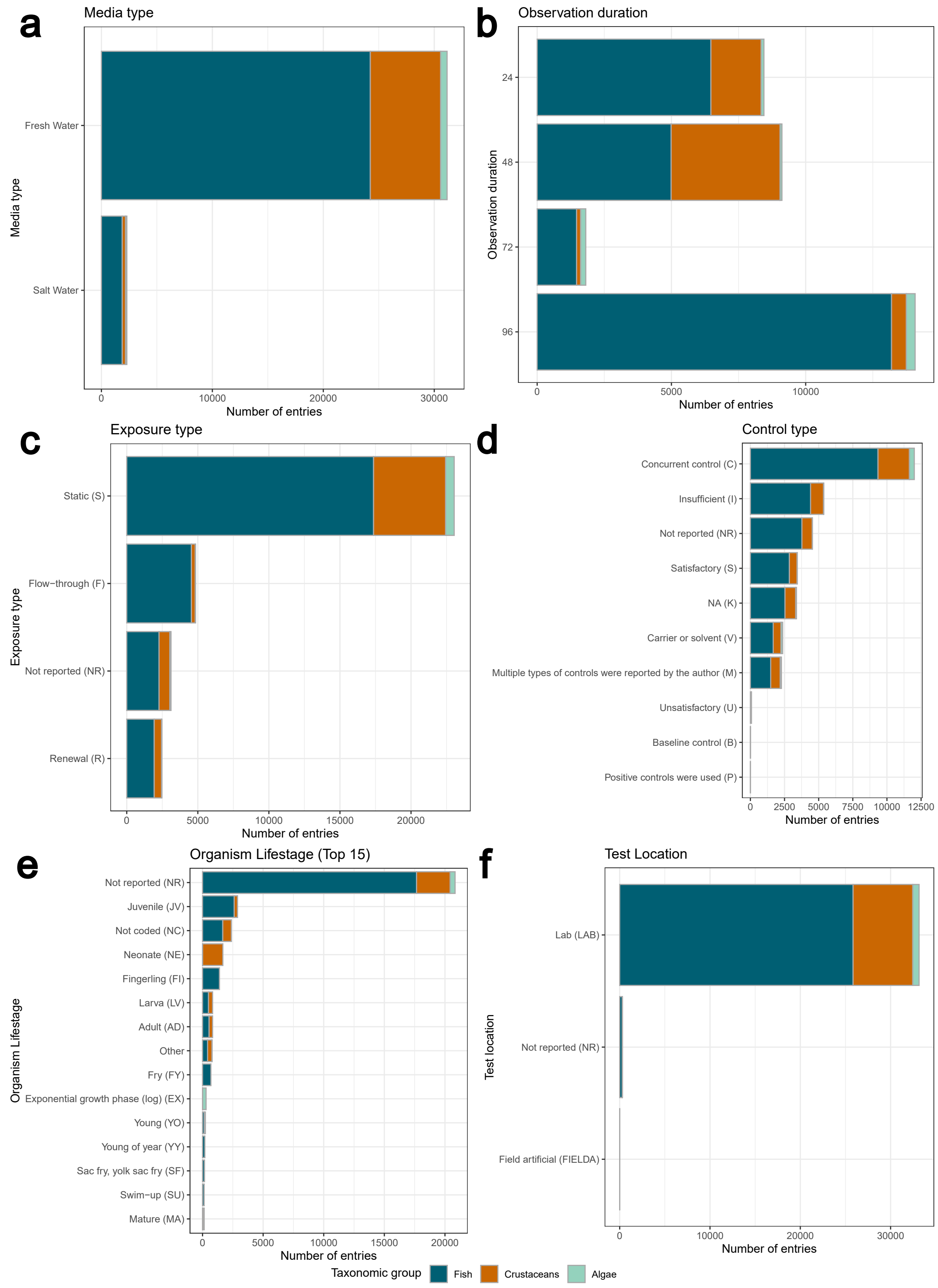

Figure 2. Overview of the most common values for experimental properties parameters. a: Media type; b: Observation duration; c: Exposure type; d: Control type; e: Organism life stage (only the top 15 of 48 total, less-abundant life stages are combined into "other"); f: Test location.

3 Taxonomic data

3.1 Overview

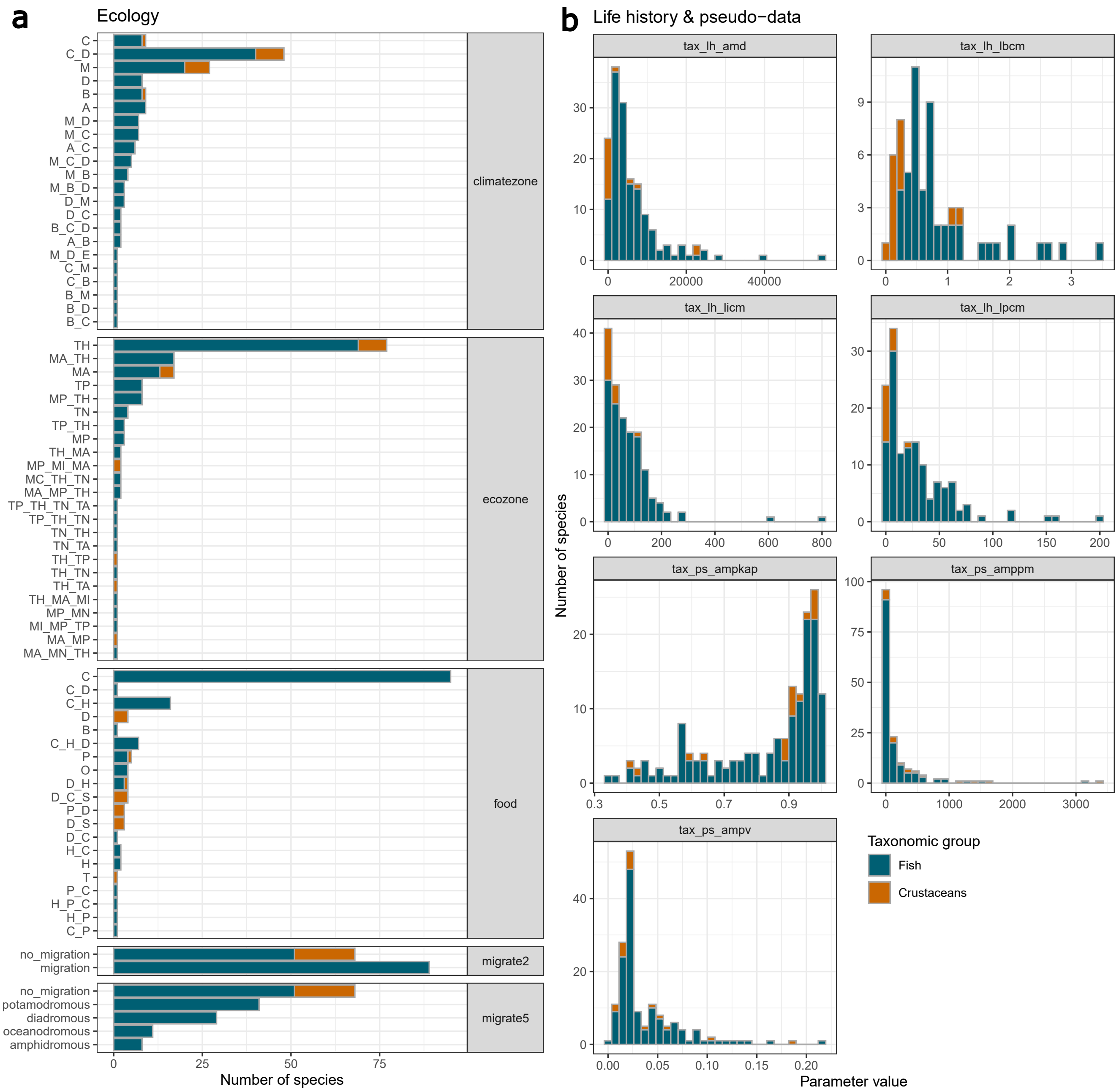

Figure 3. a: Overview of the taxonomic properties for ecology. b: Overview for life history and pseudo-data parameter values. The encodings for the different levels are given in Supplementary Tables 3-6.

3.2 Ecological data encoding

Here, we provide the encodings for the ecological data. For some parameters, we had to reduce the complexity of the encodings. More detailed descriptions of the parameters and the original encoding can be found in the Add my Pet collection

3.2.1 Climate zone

Table 3. Climate zone definitions and encodings.

Encoding	Definition
A	Tropical (megathermal) climates: every month of the year with an average temperature of 18 °C or higher, with significant precipitation
B	Dry (arid and semi-arid) climates: little precipitation
C	Temperate (mesothermal) climates: the coldest month averaging between 0 and 18 °C and at least one month averaging above 10 °C
D	Continental (microthermal) climates: at least one month averaging below -3 °C and at least one month averaging above 10 °C
E	Polar and alpine (montane) climates: every month of the year with an average temperature below 10 °C
M	Marine climates

3.2.2 Ecozone

Table 4. Ecozone definitions and encodings.

Encoding	Definition
MA	Atlantic ocean
MC	Circumglobal oceans
MI	Indian ocean
MN	Arctic ocean
MP	Pacific ocean
MS	Southern ocean
TA	Australasia (including New Guinea, New Zealand)
TH	Holarctic
TN	Neotropic (including Central America, and the Caribbean)
TP	Paleotropic

3.2.3 Migratory behavior

Table 5. Migratory definitions and encodings for 2 and 5 levels.

2-level encoding	5-level encoding	Definition
no_migration	no_migration	Do not migrate
migration	amphidromous	Migrate from fresh water to the sea, or vice versa, but not for breeding
migration	diadromous	Migrate between the sea and fresh water
migration	oceanodromous	Live and migrate wholly in the sea
migration	potamodromous	Live and migrate wholly within fresh water

3.2.4 Food

Table 6. Food definitions and encodings.

Encoding	Definition
B	Bacterivore (including micro-organisms)
C	Carnivore (living animals)
D	Detrivore (bacteria, small fungi, organic matter)
H	Herbivore (plants)
O	Omnivore (plants/animals/fungi)
P	Planktivore (small aquatic organisms, macro-movement controlled by flow, not by swimming)
S	Scavenger (dead animals)
T	Parasitic (animal tissue)

4 Chemical data

4.1 Tanimoto similarity

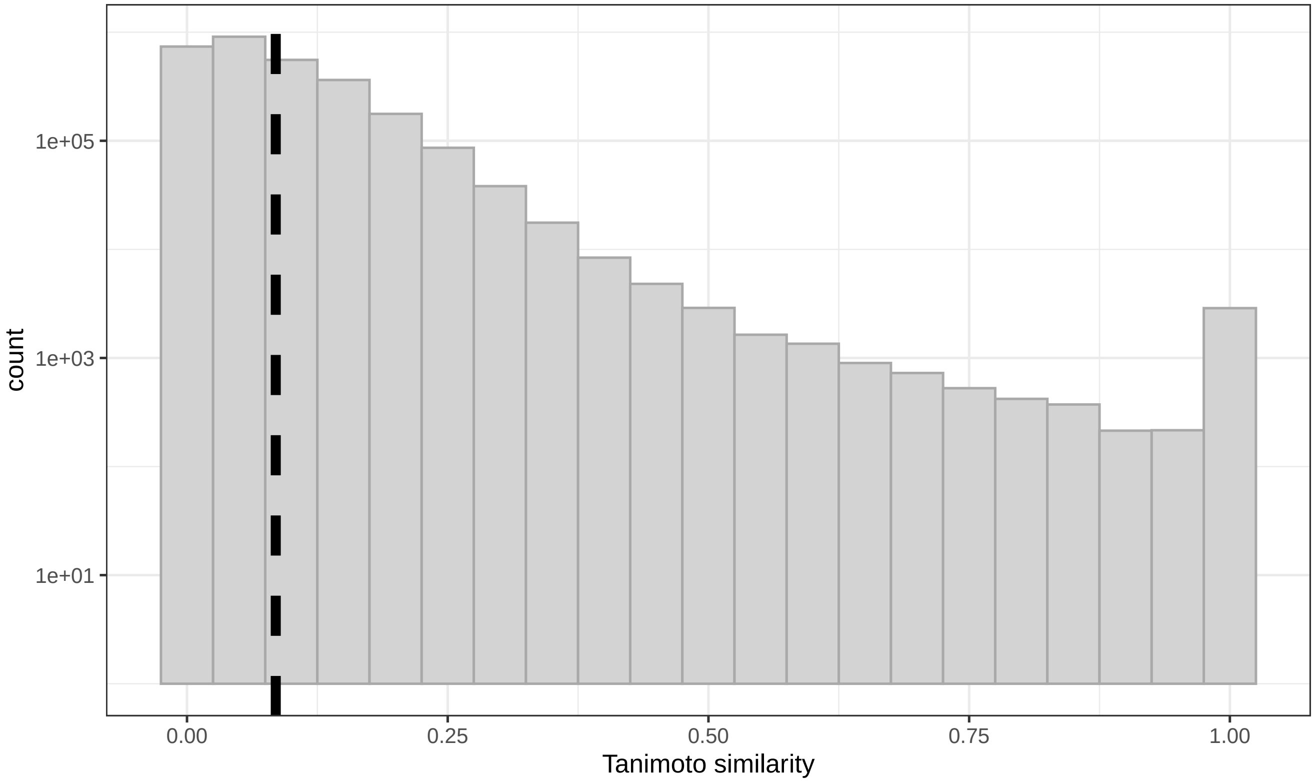

Figure 4. Histogram of the Tanimoto similarities for the chemicals in our dataset, ranging from 0 (dissimmilar) to 1 (equal). The dashed vertical line indicates the mean similarity of 0.085.

4.2 Chemical ontology

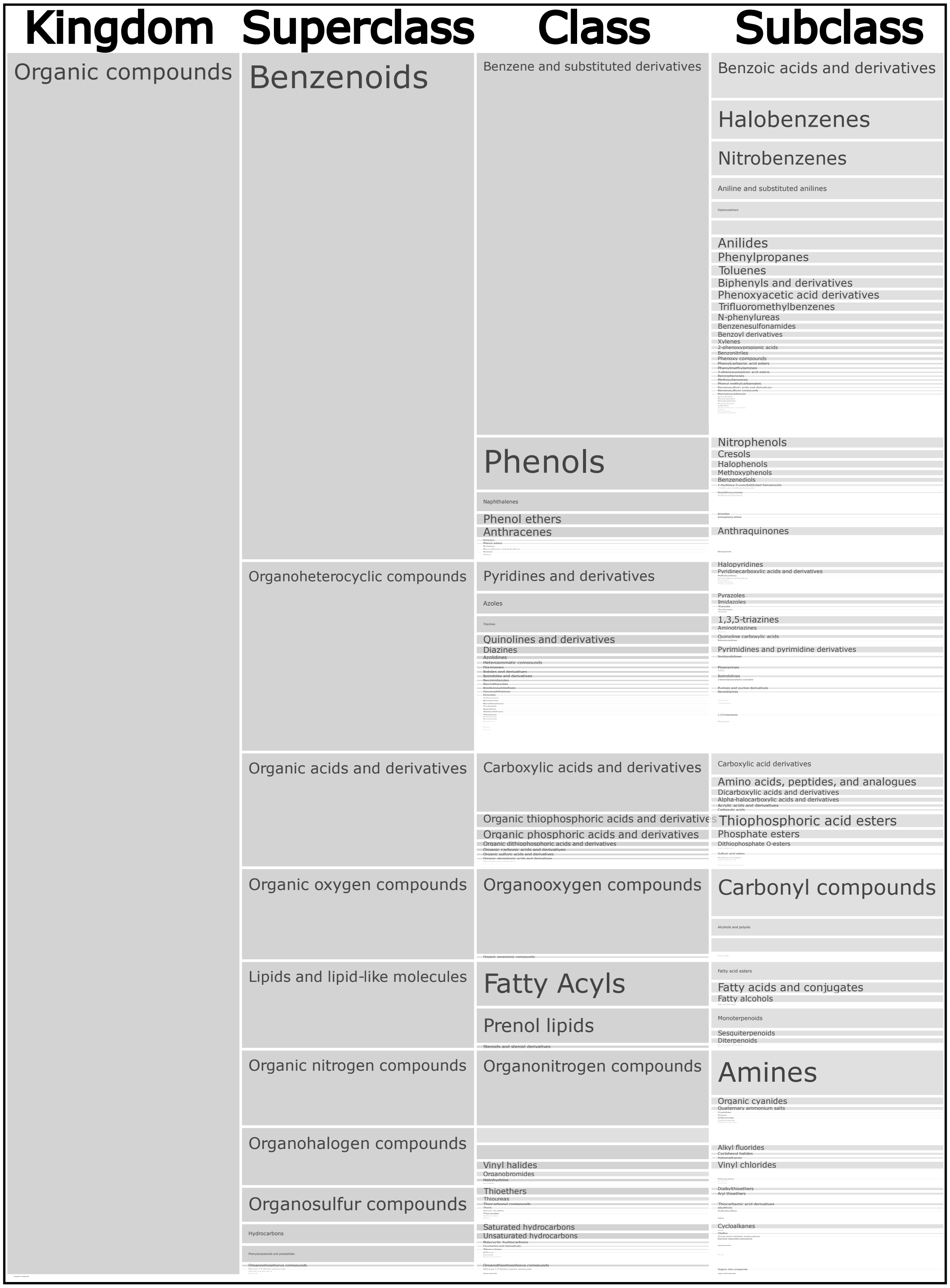

Figure 5. Icicle chart representing the chemical ontology for 2,250 of the 2,408 chemicals in our dataset, with the hierarchical levels kingdom, superclass, class, and subclass.

4.3 Chemical properties

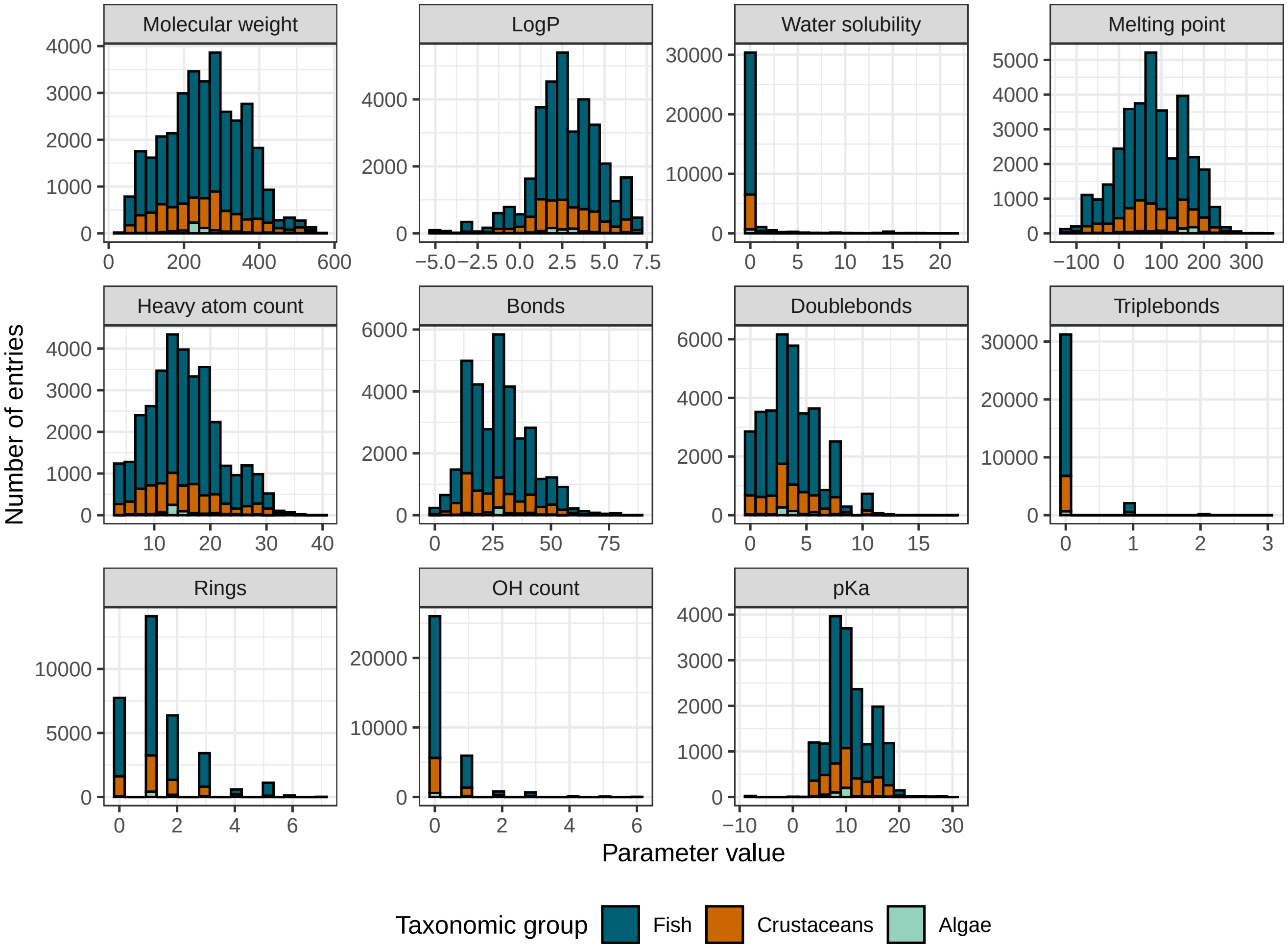

Figure 6. Overview of the distributions of the chemical properties represented in our dataset. See Table 5 in the main manuscript for more details.

4.4 Functional use categories

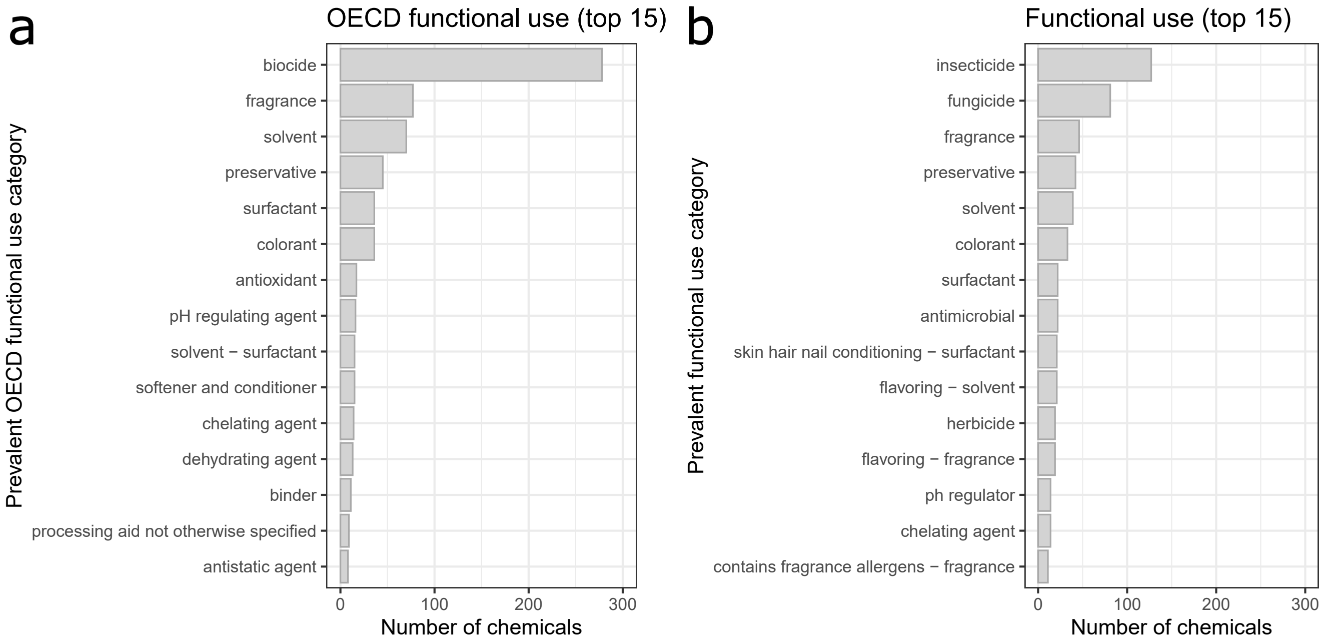

Figure 7. Prevalent functional use categories for 570 of the 2,408 chemicals in our dataset. Most chemicals have several reported uses of which we select the prevalent ones, *i.e.,* those reported in at least 20% of the cases for each chemical. a: Internationally harmonized OECD functional uses, b: non-harmonized functional uses distinguish between the different biocides.

4.5 Molecular scaffolds

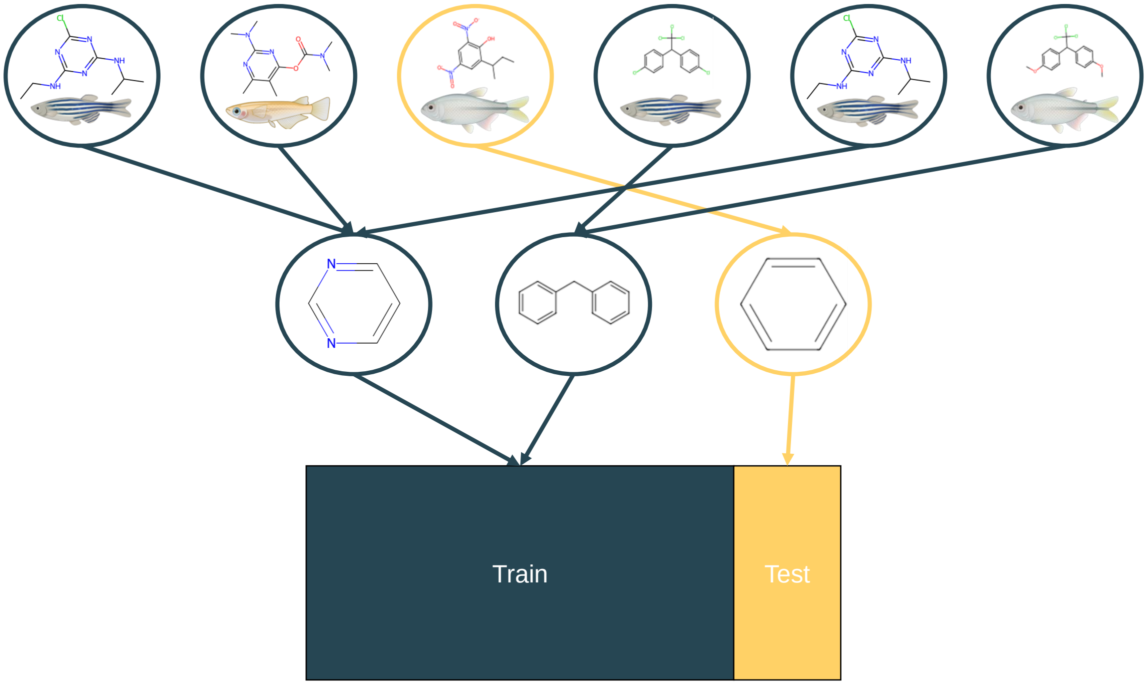

Figure 8. Schematic visualization of stratified data splitting according to the molecular scaffold. In the first step, all molecules that share a molecular scaffold are grouped together. Then, these groups are distributed into a training and a test set. This procedure reduces data leakage by ensuring that similar chemicals - defined by a common molecular scaffold - do not occur both in the train and test set.

Table 7. Canonical SMILES and chemical structures of the most common Murcko scaffolds. We do not include "no scaffold" here.

Canonical SMILES	Structure
c1ccccc1	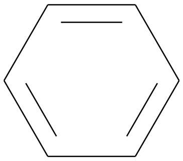
c1ccc(Cc2ccccc2)cc1	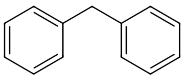
c1ccncc1	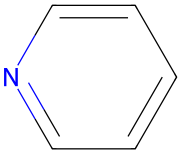

Table 7 continued from previous page

Canonical SMILES	Structure
c1ccc2ccccc2c1	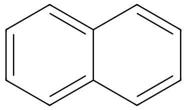
O=C(OCc1cccc(Oc2ccccc2)c1)C1CC1	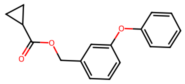
C1=CC2CC1C1C3CC(C4OC34)C21	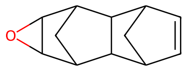
C1CCCCC1	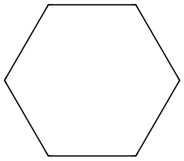
O=C(Nc1ccccc1)c1ccccc1	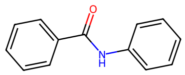
O=c1[nH]nnc2ccccc12	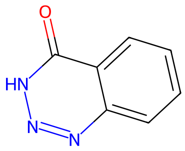
O=S1OCC2C3C=CC(C3)C2CO1	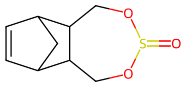
c1ncncn1	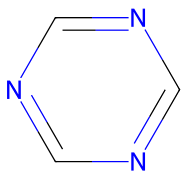

Table 7 continued from previous page

Canonical SMILES	Structure
c1cncnc1	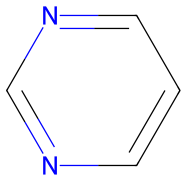
O=C1c2ccc3c(c2OC2COc4ccccc4C12)CCO3	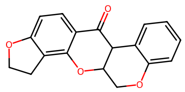
O=C(Cc1ccccc1)OCc1cccc(Oc2ccccc2)c1	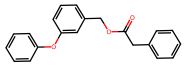
c1ccc2[nH]cnc2c1	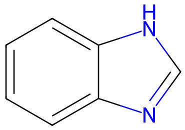
C1=NCCN1Cc1cccnc1	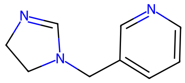
c1ccc(Oc2ccccc2)cc1	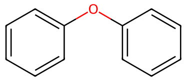
c1cc(-c2cc[nH+]cc2)cc[nH+]1	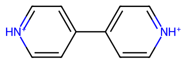
O=c1cccnn1-c1ccccc1	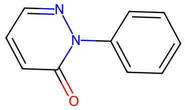

Table 7 continued from previous page

Canonical SMILES	Structure
c1ncnc(NC2CC2)n1	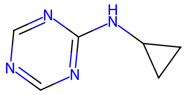
C(=Cc1ccccc1)C(C=Cc1ccccc1)=NNC1=NCCCN1	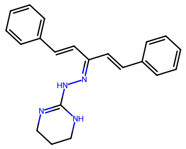
O=C(Nc1ncncn1)NS(=O)(=O)c1ccccc1	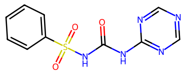
c1ccc(C2OC2(Cn2cncn2)c2ccccc2)cc1	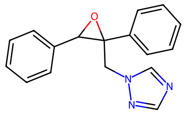
c1cc[n+]2c(c1)-c1cccc[n+]1CC2	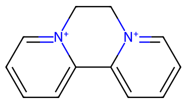
c1ccc(C2(Cn3cncn3)OCCO2)cc1	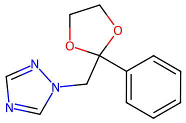
